# Transcriptomic study in women with trisomy 21 identifies a possible role of the GTPases of the immunity-associated proteins (GIMAP) in the protection of breast cancer

**DOI:** 10.1038/s41598-020-66469-w

**Published:** 2020-06-10

**Authors:** André Mégarbané, David Piquemal, Anne-Sophie Rebillat, Samantha Stora, Fabien Pierrat, Roman Bruno, Florian Noguier, Clotilde Mircher, Aimé Ravel, Marie Vilaire-Meunier, Sophie Durand, Gérard Lefranc

**Affiliations:** 1 Institut Jérôme Lejeune, CRB BioJeL, Paris, France; 2 Faculty of Medical Sciences, Lebanese University, Beirut, Lebanon; 3 Acobiom, Montpellier, France; 4 Institut de Génétique Humaine, UMR 9002 CNRS-Université de Montpellier, Montpellier, France

**Keywords:** Cancer, Genetics, Molecular biology, Oncology

## Abstract

Background: People with trisomy 21 (T21) are predisposed to developing hematological tumors, but have significantly lower-than-expected age-adjusted incidence rates of having a solid tumor. Material and methods: To identify novel genetic factors implicated in the lower breast cancer (BC) frequency observed in women with T21 than in the general population, we compared the transcriptome pattern of women with a homogeneous T21, aged more than 30 years, with or without BC, and tumoral BC tissue of control women with a normal karyotype from the study of Varley *et al*. (2014). Results: Differential analysis of gene expression between the 15 women in the T21 without BC group and BC patients in the other groups (two women with T21 and fifteen control women, respectively) revealed 154 differentially expressed genes, of which 63 were found to have similar expression profile (up- or downregulated). Of those 63 genes, four were in the same family, namely *GIMAP4*, *GIMAP6*, *GIMAP7* and *GIMAP8*, and were strongly upregulated in the T21 without BC group compared to the other groups. A significant decrease in mRNA levels of these genes in BC tissues compared to non-tumor breast tissues was also noted. Conclusion: We found that the expression of some *GIMAPs* is significantly higher in women with T21 without BC than in patients with sporadic BC. Our findings support the hypothesis that GIMAPs may play a tumor-suppressive role against BC, and open the possibility that they may also have the same role for other solid tumors in T21 patients. The search for new prognostic factors and hopefully new therapeutic or preventive strategies against BC are discussed.

## Introduction

Trisomy 21 (T21) or Down syndrome is a common genetic disorder that results from the presence of all or part of an extra chromosome 21. It is one of the most frequent and most recognizable form of intellectual disability, appearing in approximately one out of every 700 to 2000 newborns. More than 100 features of people with T21 have been described, encompassing physical, medical, and psychological features^1^.

Epidemiological studies have shown that people with T21 are more predisposed to developing hematological tumors than the general population, yet their risk of developing solid tumors is at least 12 times lower^2–8^. For instance, breast cancer (BC) is almost absent in women with T21^8–10^, even though this group shows a higher prevalence of known risk factors for BC, such as nulliparity, higher body mass index and obesity rates, sedentary life, increased chromosomal instability, dysfunctional mitochondria, overexpression of several known oncogenes and attenuation of known tumor suppressors, premature aging, DNA repair anomalies, immune deficiency, and excess of thoracic X-rays for recurrent respiratory infections^6,11^. Environmental protective factors such as reduced estrogen exposure, early menopause, or no alcohol use^10^ are not sufficient to explain this paradox.

However, BC incidence in women with intellectual disability that have many risk patterns as women with T21 is similar to that observed in the general population^8^. This suggests that genetic factors might protect women with T21 against BC^12^. Although many tumor repressor genes are localized on chromosome 21^6,12,13^, having three copies of chromosome 21 is unlikely to be the only explanation for the protection of women with T21 from BC^6^.

We hypothesized that other, as yet unknown factors or pathways could be responsible for protecting women with T21 from BC. To identify these genes, we compared the transcriptomes of women with T21 with or without BC to those of women from the general population with or without BC.

## Materials and Methods

## Patients

More than 5500 clinical files of T21 patients collected at the Jérôme Lejeune Institute (Paris, France), were screened to identify women with a homogeneous T21 with no mosaicism or translocation, aged more than 34 years, and without chronic medication and social problems. Women who did not have BC or any mammary lesion were registered under the subgroup T21-BCF (BCF for Breast Cancer Free); women who had BC were registered under the subgroup T21-BC. No history of BC was noted in the families of these women, and no pathogenic or likely pathogenic variants in genes involved in BC were present in the T21-BC group. Written informed consent from parents or guardians on behalf of the participants was obtained prior to the study.

RNA was extracted from peripheral blood mononuclear cells (PBMCs) using a Qiagen Kit (Qiagen GmBH, Hilden, Germany).

## Sequencing and Bioinformatics

Individual differential gene expression profiles were established according to the TruSeq Stranded mRNA protocol (Illumina, CA, USA). After sequencing the RNAseq libraries on the NextSeq (Illumina) platform by Paired-End sequencing (2 × 75 bp), cleaning and trimming of sequences were executed with Trimmomatic software (V0.37), and quality control was checked with the FastQC software. Sequences were then mapped onto the human genome with TopHat2 and aligned reads for each gene were counted with the HTSEQ-count software. Finally, genes were annotated with the GRCh38 human genome version.

To obtain transcriptomic data of women without T21, 30 RNAseq libraries were analyzed. Fifteen were from the tumoral tissue of women with a normal karyotype and with sporadic BC, called C-BC (C for Control), from the study of Varley *et al*. (RunID: SRR1313090, SRR1313095, SRR1313096, SRR1313098, SRR1313099, SRR1313104, SRR1313105, SRR1313111, SRR1313112, SRR1313115, SRR1313116, SRR1313119, SRR1313120, and SRR1313122, SRR1313128)^14^. The other 15 RNAseq libraries (RNA extracted from PBMC) were from healthy women without BC (C-BCF) incorporated from the Matrix of RNAseq database that focuses on the RNAseq method, which reflects the expression of the genes in a specific condition, and contains 21,800 human RNAseq profiles. The relative log expression (RLE) strategy^15^ was chosen as the strategy for normalization.

## Differential Expression Analysis

We performed a differential expression analysis between the T21-BCF versus the T21-BC, C-BC, and C-BCF libraries with R software version 3.4.1 and the edgeR package v3.20.1. Selection parameters were an FDR value less than 0.001, a logFC value less than −1 or greater than +1, and a logCPM greater than 5. These stringent parameters were chosen to account for the intrinsic differences between the samples, which were derived from different tissues (PBMCs or breast tumor tissue).

### Ethical approval and consent to participate

This study conformed to the tenets of the Declaration of Helsinki and was approved by the Institute Jérôme Lejeune Committee on Clinical Investigation.

## Results

**Figure 1:**
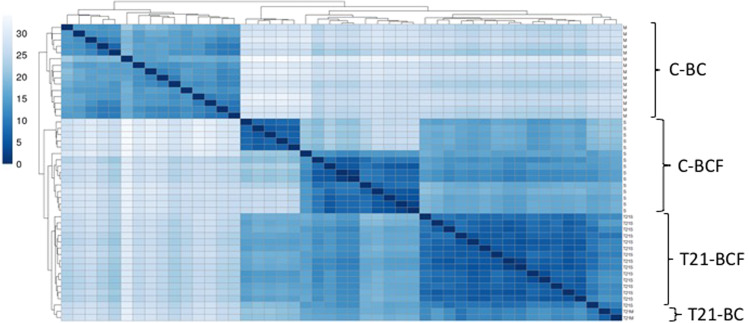
A heatmap of the distance matrix showing the similarities and dissimilarities between samples (T21-BCF; T21-BC; C-BCF; and C-BC). The normalized data are used in this figure for sample clustering. Distance between C-BC and T21-BC is the greater indicating the maximum dissimilarities between samples.

Seventeen women with T21, aged 32 to 57 years, were recruited for this study. Of these, 15 did not have BC or any mammary lesion (T21-BCF), while two had BC (T21-BC). From the 17 T21 RNAseq libraries sequenced, 485 million sequences were obtained, of which 93% were validated and mapped to the human genome (version GRCh38) (Supplementary Table 1). In the final count matrix, 57,245 different transcripts were identified. These libraries were compared to existing RNAseq libraries representing women with a normal karyotype and either with BC (C-BC) or without BC (C-BCF). The C-BC and C-BCF groups contain data from 15 samples each. A heat map to overview the similarities and dissimilarities between samples was constructed (Fig. 1). All groups were found to be well structured and homogeneous except the C-BCF group. This latter group was thus eliminated from the study, to avoid introducing bias into the analysis.

**Figure 2:**
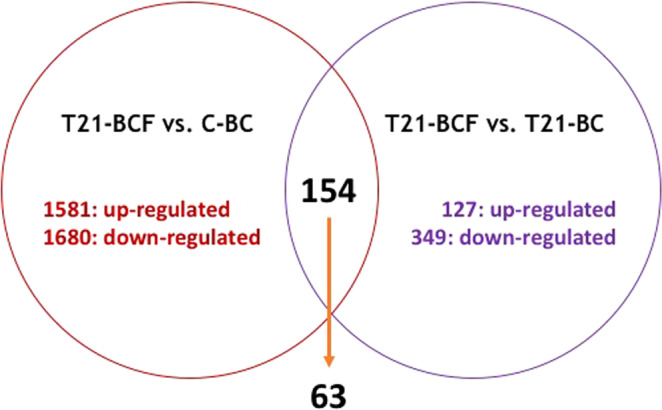
Differential analysis of gene expression between T21-BCF versus C-BC (group (**A**) and between T21-BCF versus T21-BC (group B) showing the number of genes that were differentially expressed. At the intersection of the 2 groups, 154 genes were differentially expressed, of which 63 genes had a similar expression profile (15 up- and 48 down-regulated).

Differential analysis of gene expression between T21-BCF versus C-BC revealed a total of 3,261 genes (comparison group A) that were differentially expressed. Among them, 1,581 genes were up regulated in the T21-BCF cohort versus C-BC, and 1,680 were downregulated. Between T21-BCF and T21-BC (comparison group B), a total of 476 genes were differentially expressed, with 127 genes up regulated and 349 down regulated in T21-BCF patients relative to T21-BC patients (Fig. 2).

In the T21-BCF group, we looked at the expression of genes on chromosome 21, especially those that might predispose to BC or have a positive action against BC occurrence, such as *BTG3* (OMIM 605673), *TIAM1* (OMIM 600687), *ETS2* (OMIM 164740), *DYRK1A* (OMIM 600855), *SIM2* (OMIM 600892), *Col18A1* (OMIM 120328), *IFNAR1* (OMIM 107450), *NRIP1* (OMIM 602490), *S100B* (OMIM 176990), *ERG* (OMIM 165080),and *RUNX1 (OMIM 151385*)^6^. All these genes were expressed in the T21-BCF group, but no particular or significant differential expression relative to the potential role of each gene was found.

We then examined the expression of genes in pathways that play an important role in BC development, in particular those involved in reducing apoptosis allowing proliferation, or those that have been linked to diagnosis, prognosis and hereditary susceptibility (KEGG database; Kyoto Encyclopedia of Genes and Genomes: www.kegg.jp/kegg/kegg1.html). Specifically, we studied the PI3K/AKT/mTOR, EGFR, ovarian steroidogenesis, tyrosine kinase inhibitor resistance, RAP1 thyroid hormone signaling, parathyroid hormone synthesis, GNRH signaling, prolactin signaling pathways, and immune pathways. In all of them, different transcript levels were noted in some of the genes, but nothing obvious to conclude to a particular alteration of a signaling pathway.

**Figure 3:**
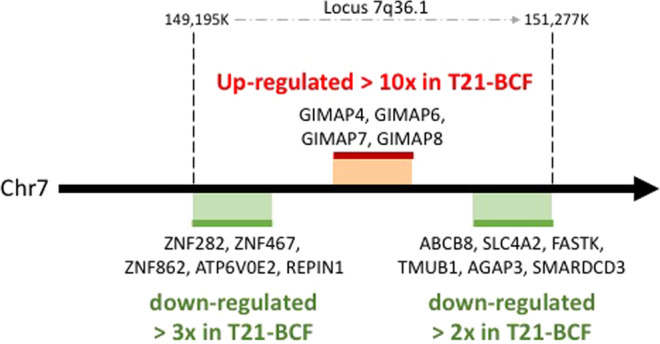
Physical map of 7q36.1 locus with groups of co-regulated genes in differential analysis between T21-BCF compared to the T21-BC or C-BC groups.

We thus compared transcript expression between 2 different groups: T21-BCF versus C-BC (group A) and T21-BCF versus T21-BC (group B). At the intersection of comparison groups A and B, 154 differentially expressed genes were identified. Among them, 63 genes were found to have the same regulation profile (15 up- and 48 down-regulated) (Table 1). Among the identified genes, a group of four genes in the GTPases of the immunity-associated proteins family (*GIMAP)* – *GIMAP4*, *GIMAP6*, *GIMAP7* and *GIMAP8* – were strongly upregulated (fold ration >10) in the T21-BCF group compared to the T21-BC or C-BC patients (Fig. 3).

**Table 1:** List of the 63 genes showing the same regulation profile between groups A (T21-BCF versus C-BC) and B (T21-BCF versus T21-BC).

Gene Name	Ensembl Id.	T21-BCF vs C-BC ratio	T21-BCF vs T21-BC ratio	Gene description
THEMIS	ENSG00000172673	88.6	6.9	Thymocyte selection associated
CD3G	ENSG00000160654	85.0	7.4	CD3g molecule, gamma (CD3-TCR complex); The CD3 complex mediates signal transduction
TRAC	ENSG00000277734	40.4	10.9	Nuclear receptor corepressor 2; Transcriptional corepressor. Mediates the transcriptional repression activity of some nuclear receptors by promoting chromatin condensation, thus preventing access of the basal transcription. Isoform 1 and isoform 5 have different affinities for different nuclear receptors. Involved in the regulation BCL6-dependent of the germinal center (GC) reactions, mainly through the control of the GC B-cells proliferation and survival
GIMAP4	ENSG00000133574	15.9	4.7	GTPase, IMAP family member 4; May play a role in regulating lymphocyte apoptosis (By similarity). Exhibits intrisinic GTPase activity. Shows a higher affinity for GDP over GTP (about 12-fold higher), and binding shows an absolute requirement for magnesium
GIMAP6	ENSG00000133561	13.7	6.9	GTPase, IMAP family member 6
GIMAP7	ENSG00000179144	10.9	9.5	GTPase, IMAP family member 7; The dimer has GTPase activity; the active site contains residues from both subunits
GIMAP8	ENSG00000171115	10.4	6.6	GTPase, IMAP family member 8; Exerts an anti-apoptotic effect in the immune system and is involved in responses to infections
MAT2B	ENSG00000038274	6.0	3.9	Methionine adenosyltransferase II, beta; Non-catalytic regulatory subunit of S-adenosylmethionine synthetase 2 (MAT2A), an enzyme that catalyzes the formation of S- adenosylmethionine from methionine and ATP. Regulates the activity of S-adenosylmethionine synthetase 2 by changing its kinetic properties, rendering the enzyme more susceptible to S- adenosylmethionine inhibition
LPIN2	ENSG00000101577	5.9	2.9	Lipin 2; Plays important roles in controlling the metabolism of fatty acids at differents levels. Acts as a magnesium-dependent phosphatidate phosphatase enzyme which catalyzes the conversion of phosphatidic acid to diacylglycerol during triglyceride, phosphatidylcholine and phosphatidylethanolamine biosynthesis in the reticulum endoplasmic membrane. Acts also as a nuclear transcriptional coactivator for PPARGC1A to modulate lipid metabolism (By similarity)
ATP8B2	ENSG00000143515	3.9	3.9	ATPase, aminophospholipid transporter, class I, type 8B, member 2; Catalytic component of a P4-ATPase flippase complex which catalyzes the hydrolysis of ATP coupled to the transport of aminophospholipids from the outer to the inner leaflet of various membranes and ensures the maintenance of asymmetric distribution of phospholipids. Phospholipid translocation seems also to be implicated in vesicle formation and in uptake of lipid signaling molecules (Probable)
LINS1	ENSG00000140471	3.8	2.9	Lines homolog (Drosophila)
RPA2	ENSG00000117748	3.4	2.7	Replication protein A2, 32 kDa; As part of the heterotrimeric replication protein A complex (RPA/RP-A), binds and stabilizes single-stranded DNA intermediates, that form during DNA replication or upon DNA stress. It prevents their reannealing and in parallel, recruits and activates different proteins and complexes involved in DNA metabolism. Thereby, it plays an essential role both in DNA replication and the cellular response to DNA damage. In the cellular response to DNA damage, the RPA complex controls DNA repair and DNA damage checkpoint activation. Through recruitment of ATRIP activ […]
SYNRG	ENSG00000275066	3.3	3.2	Synergin, gamma; May play a role in endocytosis and/or membrane trafficking at the trans-Golgi network (TGN). May act by linking the adapter protein complex AP-1 to other proteins
DENND2D	ENSG00000162777	3.1	2.7	DENN/MADD domain containing 2D; Guanine nucleotide exchange factor (GEF) which may activate RAB9A and RAB9B. Promotes the exchange of GDP to GTP, converting inactive GDP-bound Rab proteins into their active GTP- bound form
CRLF3	ENSG00000176390	3.1	2.8	Cytokine receptor-like factor 3; May play a role in the negative regulation of cell cycle progression
ZNF281	ENSG00000162702	0.50	0.3	Zinc finger protein 281; Transcription repressor that plays a role in regulation of embryonic stem cells (ESCs) differentiation. Required for ESCs differentiation and acts by mediating autorepression of NANOG in ESCs: binds to the NANOG promoter and promotes association of NANOG protein to its own promoter and recruits the NuRD complex, which deacetylates histones. Not required for establishement and maintenance of ESCs (By similarity). Represses the transcription of a number of genes including GAST, ODC1 and VIM. Binds to the G- rich box in the enhancer region of these genes
CPEB2	ENSG00000137449	0.49	0.2	Cytoplasmic polyadenylation element binding protein 2; May play a role in translational regulation of stored mRNAs in transcriptionally inactive haploid spermatids. Binds to poly(U) RNA oligomers (By similarity)
WDFY3	ENSG00000163625	0.49	0.3	WD repeat and FYVE domain containing 3; Required for selective autophagy (aggrephagy) but not for autophagic degradation of bulk cytospol in response to starvation. Involved in the formation and degradation of cytoplasmic polyubiquitin-containing bodies (p62 bodies, ALIS/aggresome-like induced structures). May play a role as adaptor or scaffolding protein by promoting the association of the E3-like ligase ATG12-ATG5-ATG16L and LC3 to ubiquitinated target substrate. The association with GABARAP is required for its recruitment to LC3B-positive p62 bodies suggesting a role in targeting ce […]
PLEKHM2	ENSG00000116786	0.49	0.3	Pleckstrin homology domain containing, family M (with RUN domain) member 2; May play a role in the regulation of conventional kinesin activity. Required for maintenance of the Golgi apparatus organization. May play a role in membrane tubulation
TOM1	ENSG00000100284	0.48	0.3	Target of myb1 (chicken); May be involved in intracellular trafficking. Probable association with membranes
LRP1	ENSG00000123384	0.46	0.3	Low density lipoprotein receptor-related protein 1; Endocytic receptor involved in endocytosis and in phagocytosis of apoptotic cells. Required for early embryonic development. Involved in cellular lipid homeostasis. Involved in the plasma clearance of chylomicron remnants and activated LRPAP1 (alpha 2-macroglobulin), as well as the local metabolism of complexes between plasminogen activators and their endogenous inhibitors. May modulate cellular events, such as APP metabolism, kinase-dependent intracellular signaling, neuronal calcium signaling as well as neurotransmission
SLC35B2	ENSG00000157593	0.44	0.3	Solute carrier family 35, member B2; Mediates the transport of adenosine 3′-phospho 5′- phosphosulfate (PAPS), from cytosol into Golgi. PAPS is a universal sulfuryl donor for sulfation events that take place in the Golgi. May indirectly participate in activation of the NF- kappa-B and MAPK pathways
ODF3B	ENSG00000177989	0.43	0.2	Outer dense fiber of sperm tails 3B
UBXN11	ENSG00000158062	0.42	0.4	UBX domain protein 11; May be involved in the reorganization of actin cytoskeleton mediated by RND1, RND2 AND RND3. Promotes RHOA activation mediated by GNA12 and GNA13 (By similarity)
FOXO3	ENSG00000118689	0.42	0.3	Forkhead box O3; Transcriptional activator which triggers apoptosis in the absence of survival factors, including neuronal cell death upon oxidative stress. Recognizes and binds to the DNA sequence 5′-[AG]TAAA[TC]A-3′. Participates in post-transcriptional regulation of MYC: following phosphorylation by MAPKAPK5, promotes induction of miR-34b and miR-34c expression, 2 post- transcriptional regulators of MYC that bind to the 3′UTR of MYC transcript and prevent its translation
DOT1L	ENSG00000104885	0.41	0.4	DOT1-like, histone H3 methyltransferase (S. cerevisiae); Histone methyltransferase. Methylates ‘Lys-79’ of histone H3. Nucleosomes are preferred as substrate compared to free histones. Binds to DNA
SKIL	ENSG00000136603	0.40	0.2	SKI-like oncogene; May have regulatory role in cell division or differentiation in response to extracellular signals
SNX9	ENSG00000130340	0.37	0.2	Sorting nexin 9; Involved in endocytosis and intracellular vesicle trafficking, both during interphase and at the end of mitosis. Required for efficient progress through mitosis and cytokinesis. Required for normal formation of the cleavage furrow at the end of mitosis. Plays a role in endocytosis via clathrin-coated pits, but also clathrin-independent, actin-dependent fluid-phase endocytosis. Plays a role in macropinocytosis. Promotes internalization of TNFR. Promotes degradation of EGFR after EGF signaling. Stimulates the GTPase activity of DNM1. Promotes DNM1 oligomerization. Promot […]
BCL6	ENSG00000113916	0.37	0.2	B-cell CLL/lymphoma 6; Transcriptional repressor mainly required for germinal center (GC) formation and antibody affinity maturation which has different mechanisms of action specific to the lineage and biological functions. Forms complexes with different corepressors and histone deacetylases to repress the transcriptional expression of different subsets of target genes. Represses its target genes by binding directly to the DNA sequence 5′-TTCCTAGAA-3′ (BCL6- binding site) or indirectly by repressing the transcriptional activity of transcription factors. In GC B-cells, represses genes t […]
TP53INP2	ENSG00000078804	0.37	0.1	Tumor protein p53 inducible nuclear protein 2; Dual regulator of transcription and autophagy. Positively regulates autophagy and is required for autophagosome formation and processing. May act as a scaffold protein that recruits MAP1LC3A, GABARAP and GABARAPL2 and brings them to the autophagosome membrane by interacting with VMP1 where, in cooperation with the BECN1-PI3-kinase class III complex, they trigger autophagosome development. Acts as a transcriptional activator of THRA
RIPOR1	ENSG00000039523	0.36	0.4	Poliovirus receptor-related 2 (herpesvirus entry mediator B); Probable cell adhesion protein
MAFG	ENSG00000197063	0.35	0.2	V-maf musculoaponeurotic fibrosarcoma oncogene homolog G (avian); Since they lack a putative transactivation domain, the small Mafs behave as transcriptional repressors when they dimerize among themselves. However, they seem to serve as transcriptional activators by dimerizing with other (usually larger) basic-zipper proteins and recruiting them to specific DNA-binding sites. Small Maf proteins heterodimerize with Fos and may act as competitive repressors of the NF-E2 transcription factor. Transcription factor, component of erythroid-specific transcription factor NF- E2. Activates glob […]
PEA15	ENSG00000162734	0.35	0.4	Phosphoprotein enriched in astrocytes 15; Blocks Ras-mediated inhibition of integrin activation and modulates the ERK MAP kinase cascade. Inhibits RPS6KA3 activities by retaining it in the cytoplasm (By similarity). Inhibits both TNFRSF6- and TNFRSF1A-mediated CASP8 activity and apoptosis. Regulates glucose transport by controlling both the content of SLC2A1 glucose transporters on the plasma membrane and the insulin-dependent trafficking of SLC2A4 from the cell interior to the surface
LDLR	ENSG00000130164	0.33	0.3	Low density lipoprotein receptor; Binds LDL, the major cholesterol-carrying lipoprotein of plasma, and transports it into cells by endocytosis. In order to be internalized, the receptor-ligand complexes must first cluster into clathrin-coated pits
BCL3	ENSG00000069399	0.33	0.1	B-cell CLL/lymphoma 3; Contributes to the regulation of transcriptional activation of NF-kappa-B target genes. In the cytoplasm, inhibits the nuclear translocation of the NF-kappa-B p50 subunit. In the nucleus, acts as transcriptional activator that promotes transcription of NF-kappa-B target genes. Contributes to the regulation of cell proliferation (By similarity)
ZBTB17	ENSG00000116809	0.33	0.3	Zinc finger and BTB domain containing 17; Transcription factor that can function as an activator or repressor depending on its binding partners, and by targeting negative regulators of cell cycle progression. Plays a critical role in early lymphocyte development, where it is essential to prevent apoptosis in lymphoid precursors, allowing them to survive in response to IL7 and undergo proper lineage commitment. Has been shown to bind to the promoters of adenovirus major late protein and cyclin D1 and activate transcription. Required for early embryonic development during gastrulation. R […]
IER3	ENSG00000137331	0.30	0.1	Immediate early response 3; May play a role in the ERK signaling pathway by inhibiting the dephosphorylation of ERK by phosphatase PP2A- PPP2R5C holoenzyme. Acts also as an ERK downstream effector mediating survival. As a member of the NUPR1/RELB/IER3 survival pathway, may provide pancreatic ductal adenocarcinoma with remarkable resistance to cell stress, such as starvation or gemcitabine treatment
CDK16	ENSG00000102225	0.27	0.3	Cyclin-dependent kinase 16; Protein kinase that plays a role in vesicle-mediated transport processes and exocytosis. Regulates GH1 release by brain neurons. Phosphorylates NSF, and thereby regulates NSF oligomerization. Required for normal spermatogenesis. Regulates neuron differentiation and dendrite development (By similarity). Plays a role in the regulation of insulin secretion in response to changes in blood glucose levels. Can phosphorylate CCNY at ‘Ser- 336’ (*in vitro*)
KIF13A	ENSG00000137177	0.26	0.2	Kinesin family member 13A; Plus end-directed microtubule-dependent motor protein involved in intracellular transport and regulating various processes such as mannose-6-phosphate receptor (M6PR) transport to the plasma membrane, endosomal sorting during melanosome biogenesis and cytokinesis. Mediates the transport of M6PR- containing vesicles from trans-Golgi network to the plasma membrane via direct interaction with the AP-1 complex. During melanosome maturation, required for delivering melanogenic enzymes from recycling endosomes to nascent melanosomes by creating peripheral recycling […]
ZNF598	ENSG00000167962	0.26	0.4	Zinc finger protein 598
KDM5B	ENSG00000117139	0.25	0.4	Lysine (K)-specific demethylase 5B; Histone demethylase that demethylates ‘Lys-4’ of histone H3, thereby playing a central role in histone code. Does not demethylate histone H3 ‘Lys-9’ or H3 ‘Lys-27’. Demethylates trimethylated, dimethylated and monomethylated H3 ‘Lys-4’. Acts as a transcriptional corepressor for FOXG1B and PAX9. Favors the proliferation of breast cancer cells by repressing tumor suppressor genes such as BRCA1 and HOXA5. In contrast, may act as a tumor suppressor for melanoma. Represses the CLOCK-ARNTL/BMAL1 heterodimer-mediated transcriptional activation of the core c […]
NFKB2	ENSG00000077150	0.24	0.2	Nuclear factor of kappa light polypeptide gene enhancer in B-cells 2 (p49/p100); NF-kappa-B is a pleiotropic transcription factor present in almost all cell types and is the endpoint of a series of signal transduction events that are initiated by a vast array of stimuli related to many biological processes such as inflammation, immunity, differentiation, cell growth, tumorigenesis and apoptosis. NF-kappa-B is a homo- or heterodimeric complex formed by the Rel-like domain-containing proteins RELA/p65, RELB, NFKB1/p105, NFKB1/p50, REL and NFKB2/p52. The dimers bind at kappa-B sites in th […]
CLMN	ENSG00000165959	0.24	0.3	Calmin (calponin-like, transmembrane)
TBC1D8	ENSG00000204634	0.23	0.3	TBC1 domain family, member 8 (with GRAM domain); May act as a GTPase-activating protein for Rab family protein(s)
PER2	ENSG00000132326	0.23	0.4	Period homolog 2 (Drosophila); Transcriptional repressor which forms a core component of the circadian clock. The circadian clock, an internal time- keeping system, regulates various physiological processes through the generation of approximately 24 hour circadian rhythms in gene expression, which are translated into rhythms in metabolism and behavior. It is derived from the Latin roots ‘circa’ (about) and ‘diem’ (day) and acts as an important regulator of a wide array of physiological functions including metabolism, sleep, body temperature, blood pressure, endocrine, immune, cardiovas […]
CCDC9	ENSG00000105321	0.22	0.5	Coiled-coil domain containing 9
SRC	ENSG00000197122	0.19	0.1	V-src sarcoma (Schmidt-Ruppin A-2) viral oncogene homolog (avian); Non-receptor protein tyrosine kinase which is activated following engagement of many different classes of cellular receptors including immune response receptors, integrins and other adhesion receptors, receptor protein tyrosine kinases, G protein- coupled receptors as well as cytokine receptors. Participates in signaling pathways that control a diverse spectrum of biological activities including gene transcription, immune response, cell adhesion, cell cycle progression, apoptosis, migration, and transformation. Due to f […]
VEGFA	ENSG00000112715	0.17	0.1	Vascular endothelial growth factor A; Growth factor active in angiogenesis, vasculogenesis and endothelial cell growth. Induces endothelial cell proliferation, promotes cell migration, inhibits apoptosis and induces permeabilization of blood vessels. Binds to the FLT1/VEGFR1 and KDR/VEGFR2 receptors, heparan sulfate and heparin. NRP1/Neuropilin-1 binds isoforms VEGF-165 and VEGF-145. Isoform VEGF165B binds to KDR but does not activate downstream signaling pathways, does not activate angiogenesis and inhibits tumor growth
PLXNB2	ENSG00000196576	0.17	0.3	Plexin B2; Cell surface receptor for SEMA4C, SEMA4D and SEMA4G that plays an important role in cell-cell signaling. Binding to class 4 semaphorins promotes downstream activation of RHOA and phosphorylation of ERBB2 at ‘Tyr-1248’. Required for normal differentiation and migration of neuronal cells during brain corticogenesis and for normal embryonic brain development. Regulates the migration of cerebellar granule cells in the developing brain. Plays a role in RHOA activation and subsequent changes of the actin cytoskeleton. Plays a role in axon guidance, invasive growth and cell migrati […]
SHTN1	ENSG00000187164	0.15	0.3	nvolved in the generation of internal asymmetric signals required for neuronal polarization and neurite outgrowth. Mediates netrin-1-induced F-actin-substrate coupling or ‘clutch engagement’ within the axon growth cone through activation of CDC42, RAC1 and PAK1-dependent signaling pathway, thereby converting the F-actin retrograde flow into traction forces, concomitantly with filopodium extension and axon outgrowth. Plays a role in cytoskeletal organization by regulating the subcellular localization of phosphoinositide 3-kinase (PI3K) activity at the axonal growth cone.
CTIF	ENSG00000134030	0.14	0.3	CBP80/20-dependent translation initiation factor; Specifically required for the pioneer round of mRNA translation mediated by the cap-binding complex (CBC), that takes place during or right after mRNA export via the nuclear pore complex (NPC). Acts via its interaction with the NCBP1/CBP80 component of the CBC complex and recruits the 40 S small subunit of the ribosome via eIF3. In contrast, it is not involved in steady state translation, that takes place when the CBC complex is replaced by cytoplasmic cap-binding protein eIF4E. Also required for nonsense-mediated mRNA decay (NMD), the p […]
CDC42BPB	ENSG00000198752	0.13	0.4	CDC42 binding protein kinase beta (DMPK-like); Serine/threonine-protein kinase which is an important downstream effector of CDC42 and plays a role in the regulation of cytoskeleton reorganization and cell migration. Regulates actin cytoskeletal reorganization via phosphorylation of PPP1R12C and MYL9/MLC2. In concert with MYO18A and LURAP1, is involved in modulating lamellar actomyosin retrograde flow that is crucial to cell protrusion and migration. Phosphorylates PPP1R12A
CDC42EP4	ENSG00000179604	0.11	0.2	CDC42 effector protein (Rho GTPase binding) 4; Probably involved in the organization of the actin cytoskeleton. May act downstream of CDC42 to induce actin filament assembly leading to cell shape changes. Induces pseudopodia formation, when overexpressed in fibroblasts
ST14	ENSG00000149418	0.11	0.3	Suppression of tumorigenicity 14 (colon carcinoma); Degrades extracellular matrix. Proposed to play a role in breast cancer invasion and metastasis. Exhibits trypsin-like activity as defined by cleavage of synthetic substrates with Arg or Lys as the P1 site. Involved in the terminal differentiation of keratinocytes through prostasin (PRSS8) activation and filaggrin (FLG) processing
TMEM63B	ENSG00000137216	0.10	0.3	Transmembrane protein 63B; Acts as an osmosensitive calcium-permeable cation channel
LMNA	ENSG00000160789	0.09	0.3	Lamin A/C; Lamins are components of the nuclear lamina, a fibrous layer on the nucleoplasmic side of the inner nuclear membrane, which is thought to provide a framework for the nuclear envelope and may also interact with chromatin. Lamin A and C are present in equal amounts in the lamina of mammals. Plays an important role in nuclear assembly, chromatin organization, nuclear membrane and telomere dynamics. Required for normal development of peripheral nervous system and skeletal muscle and for muscle satellite cell proliferation. Required for osteoblastogenesis and bone formation. Also […]
SPHK1	ENSG00000176170	0.08	0.1	Sphingosine kinase 1; Catalyzes the phosphorylation of sphingosine to form sphingosine 1-phosphate (SPP), a lipid mediator with both intra- and extracellular functions. Also acts on D-erythro-sphingosine and to a lesser extent sphinganine, but not other lipids, such as D,L-threo-dihydrosphingosine, N,N-dimethylsphingosine, diacylglycerol, ceramide, or phosphatidylinositol
TRAF4	ENSG00000076604	0.08	0.4	TNF receptor-associated factor 4; Adapter protein and signal transducer that links members of the tumor necrosis factor receptor (TNFR) family to different signaling pathways. Plays a role in the activation of NF-kappa-B and JNK, and in the regulation of cell survival and apoptosis. Regulates activation of NF-kappa-B in response to signaling through Toll-like receptors. Required for normal skeleton development, and for normal development of the respiratory tract (By similarity). Required for activation of RPS6KB1 in response to TNF signaling. Modulates TRAF6 functions
INSR	ENSG00000171105	0.08	0.3	Insulin receptor; Receptor tyrosine kinase which mediates the pleiotropic actions of insulin. Binding of insulin leads to phosphorylation of several intracellular substrates, including, insulin receptor substrates (IRS1, 2, 3, 4), SHC, GAB1, CBL and other signaling intermediates. Each of these phosphorylated proteins serve as docking proteins for other signaling proteins that contain Src- homology-2 domains (SH2 domain) that specifically recognize different phosphotyrosines residues, including the p85 regulatory subunit of PI3K and SHP2. Phosphorylation of IRSs proteins lead to the act […]
TRIP10	ENSG00000125733	0.05	0.1	Thyroid hormone receptor interactor 10
SERPING1	ENSG00000149131	0.05	0.1	Serpin peptidase inhibitor, clade G (C1 inhibitor), member 1; Activation of the C1 complex is under control of the C1- inhibitor. It forms a proteolytically inactive stoichiometric complex with the C1r or C1s proteases. May play a potentially crucial role in regulating important physiological pathways including complement activation, blood coagulation, fibrinolysis and the generation of kinins. Very efficient inhibitor of FXIIa. Inhibits chymotrypsin and kallikrein
CACFD1	ENSG00000160325	0.03	0.3	Calcium channel flower domain containing 1
NECTIN2	ENSG00000130202	0.01	0.1	Nuclear receptor corepressor 2; Transcriptional corepressor. Mediates the transcriptional repression activity of some nuclear receptors by promoting chromatin condensation, thus preventing access of the basal transcription. Isoform 1 and isoform 5 have different affinities for different nuclear receptors. Involved in the regulation BCL6-dependent of the germinal center (GC) reactions, mainly through the control of the GC B-cells proliferation and survival

## Discussion

To determine why BC occurs at a lower frequency in women with T21 compared to the general population, we studied the transcriptome profile of 17 women with T21: 15 without BC (T21-BCF) and 2 with BC (T21-BC), in addition to 15 control women with a normal karyotype and BC (C-BC). The comparison of transcript expression between groups identified four genes, namely, *GIMAP4*, *GIMAP6*, *GIMAP7* and *GIMAP8*, that were strongly upregulated in the T21-BCF group compared to the T21-BC or C-BC groups.

The *GIMAP* genes are located within a region on chromosome 7q35–7q36.1. They code for a family of proteins mainly expressed in the immune system. *GIMAP* genes promote immunological functions such as thymocyte development, apoptosis of peripheral lymphocytes and T helper cell differentiation. When altered these genes are linked to immunological diseases such as T cell lymphopenia, acute myeloid leukemia and autoimmune diseases^16–19^.

Interestingly, lymphocytes, including T cells, T regulatory cells, and natural killer cells, and their cytokine release patterns, are involved in both primary prevention and recurrence of BC^20^. Furthermore, it has been hypothesized that chronic inflammation involving T lymphocytes is a possible pathophysiological pathway to breast adenocarcinoma^20,21^. Latter observations lead to a possible role of *GIMAPs* in cancer. In fact, in addition to their involvement in the regulation of the immune system, *GIMAPs* might act as tumor suppressor genes, as was speculated by Krucken *et al*. who showed that all *GIMAPs* are expressed at very low levels in diverse cancer tissues and cell lines^22^. This hypothesis was further confirmed by other studies that proved that *GIMAP4* accelerates the execution of programmed cell death^15^, while *GIMAP6* plays a role in the autophagic process^23^. On the other hand, a study on non-small cell lung cancer (NSCLC) showed that the expression of *GIMAP4*, *GIMAP6*, and *GIMAP8* were lower in tumor tissues than in adjacent non-tumor tissues^24^. Interestingly, *GIMAP8* mRNA level was abnormally elevated in the adjacent non-tumor tissues compared to that in the control lung tissues^24^. Furthermore, on a recent study performed on hepatocellular carcinoma (HCC), Huang *et al*. found that the mRNA expression of *GIMAP5* and *GIMAP6* were significantly downregulated in the HCC tumor samples and in the blood samples from HCC patients in comparison to matched non-tumor tissue samples, and blood from healthy subjects^25^. GIMAP5 and GIMAP6 proteins followed the same scheme of expression. Results suggesting the involvement of *GIMAP5* and *GIMAP6* in the pathogenesis of HCC^24^.

Despite the low number of patients with T21 and BC included in our study (2 patients), our findings suggest that the overexpression of different *GIMAPs* might have a tumor repression function in women with T21. In order to further support these results and check if *GIMAPs* play also a role in BC in women in general and not only in T21 patients, we evaluated the expression of GIMAP family members in 62 RNAseq libraries of 18 women without BC, 16 with DCIS (Ductal Carcinoma *In Situ*), 13 with triple-negative BC, and 15 with HER2 (human epidermal growth factor receptor 2) positive BC from the study of Varley *et al*. (Supplementary Table 2)^14^. Interestingly, our data show a significant decrease in mRNA levels of *GIMAP4*, *GIMAP6*, *GIMAP7* and *GIMAP8* in BC tissues, compared to DCIS tissues, and non-tumor breast tissues from either women with or without BC (supplementary 2). Furthermore, the mRNA levels of those 4 *GIMAPs* were significantly downregulated in the blood samples from patients with HER2+ or Triple-negative BC. These findings align with previous research related to the association between *GIMAP* genes and cancer and suggest that these genes act as breast tumor suppressing genes, perhaps by inhibiting cell proliferation, enhancing apoptosis or controlling the cell cycle. On the other hand, the observed overexpression of the *GIMAP* genes in T21 patients might explain the paradox that although people with T21 have an increased risk of leukemia and often show immune biological abnormalities and clinical immunodeficiency, they seem to be protected against solid tumors^26^.

In our cohort of women with T21, no cases of BC were reported in the families. In contrast, in some reported women with T21 and BC, the occurrence of BC in other family members was observed. It was linked to the presence of a pathogenic mutation predisposing to BC and associated with a risk of developing BC equals to that of the general population^11^. Whether the protective action of *GIMAPs* works only in the absence of mutations in known BC-promoting genes remains to be investigated.

Recently, different studies have shown interest in IL-12 as a potential agent for anti-tumor immunotherapy^27^. This immunocytokine, that is essential for the differentiation of the Th1 lineage, was found to upregulate the *GIMAP1* and the *GIMAP4* genes, in addition to Th1 cytokines (IL-2, TNF-alpha, IFN-gamma, etc.)^17,18,28,29^. The contribution of IL-12 to cell cycle control, cell proliferation inhibition and/or apoptosis induction, might thus be mediated by the *GIMAP* genes and/or the cytokines of the Th1 lineage that are interestingly overexpressed in T21^30^.

## Conclusions

This is the first time that a transcriptomic approach was used in a cohort of women with T21 with the aim of finding BC-related genetic factors. Our study identified a higher *GIMAP* expression in women with T21 without BC compared to BC patients either with T21 or a normal karyotype. It also showed that *GIMAP* genes are downregulated in BC tissues compared to non-tumor tissues. The characterization of the molecular mechanisms leading to *GIMAP* overexpression might help to better understand the decreased incidence of BC in women with T21. A similar approach could be used to identify if GIMAPs and/or other factors are involved in other solid tumors and secondary cancers. The ultimate goal would be to search for new prognostic factors, and hopefully new therapeutic or preventive approaches against cancer.

## Supplementary information

Supplementary information.

Supplementary information2.

## Data Availability

The data and the material are available at the CRB BioJel, Jerome Lejeune Institute, Paris, France.
