# Supplementary material for: Transcriptomic study in women with trisomy 21 identifies a possible role of the GTPases of the immunity-associated proteins (GIMAP) in the protection of breast cancer
Source: Sci Rep. 2020 Jun 10;10:9447. doi: 10.1038/s41598-020-66469-w (PMC7286899; doi:10.1038/s41598-020-66469-w)
Supplement: Supplementary file 1 — Supplementary information. [file 41598_2020_66469_MOESM1_ESM.pdf]

Transcriptomic study in women with trisomy 21 identifies a possible role of the GTPases of the immunity-associated proteins (GIMAP) in the protection of breast cancer.

André Mégarbané[1,*], David Piquemal[2], Anne-Sophie Rebillat[1], Samantha Stora[1], Fabien Pierrat[2], Roman Bruno[2], Florian Noguier[2], Clotilde Mircher[1], Aime Ravel[1], Marie Vilaire-Meunier[1], Sophie Durand[1], Gérard Lefranc[3]

Library Id.	Number of sequences	Number of sequences mapped	%
T21-BCF_1	23152589	21494645	(92.84%)
T21-BCF_2	27713824	25807280	(93.12%)
T21-BCF_3	28064939	25921722	(92.36%)
T21-BCF_4	28765884	25214903	(87.66%)
T21-BCF_5	32610010	30772689	(94.37%)
T21-BCF_6	29965581	27900514	(93.11%)
T21-BCF_7	30453761	28616108	(93.97%)
T21-BCF_8	30948856	28800811	(93.06%)
T21-BCF_9	29493929	27085838	(91.84%)
T21-BCF_10	27305777	25528654	(93.49%)
T21-BCF_11	25876475	24092827	(93.11%)
T21-BCF_12	30455708	28567094	(93.80%)
T21-BCF_13	25411461	23531276	(92.60%)
T21-BCF_14	29329743	26765008	(91.26%)
T21-BCF_15	31737781	29692754	(93.56%)
T21-BC_1	26639925	24590583	(92.31%)
T21-BC_2	27486977	25448489	(92.58%)
	485 413 220	449 831 195	93%

Supplementary Table 1: Seventeen women with T21, aged 32 to 57 years, were recruited for this study. Of these, 15 did not have BC or any mammary lesion (T21-BCF), while two had BC (T21-BC). From the 17 T21 RNAseq libraries sequenced, 485 million sequences were obtained, of which 93% were validated and mapped to the human genome (version GRCh38).
