# Supplementary material for: Transcriptomic study in women with trisomy 21 identifies a possible role of the GTPases of the immunity-associated proteins (GIMAP) in the protection of breast cancer
Source: Sci Rep. 2020 Jun 10;10:9447. doi: 10.1038/s41598-020-66469-w (PMC7286899; doi:10.1038/s41598-020-66469-w)
Supplement: Supplementary file 2 — Supplementary information2. [file 41598_2020_66469_MOESM2_ESM.pdf]

Transcriptomic study in women with trisomy 21 identifies a possible role of the GTPases of the immunity-associated proteins (GIMAP) in the protection of breast cancer.

André Mégarbané[1,2 *], David Piquemal[3], Anne-Sophie Rebillat[1], Samantha Stora[1], Fabien Pierrat[3], Roman Bruno[3], Florian Noguier[3], Clotilde Mircher[1], Aime Ravel[1], Marie Vilaire-Meunier[1], Sophie Durand[1], Gérard Lefranc[4]

		log2FC	P-value
DCIS	GIMAP4	0,953	0,0157
	GIMAP6	0,108	0,7980
	GIMAP7	1,513	0,0002
	GIMAP8	0,242	0,5002
HER2+	GIMAP4	-8,6	5,15E-43
	GIMAP6	-11,9	1,25E-48
	GIMAP7	-10,1	6,31E-43
	GIMAP8	-9,56	2,33E-54
TNeg	GIMAP4	-0,70	0,1046
	GIMAP6	-2,42	1,47E-06
	GIMAP7	-0,49	0,2596
	GIMAP8	-2,29	5,15E-08

Supplementary Table 2: Differential gene expression of *GIMAP4, GIMAP6, GIMAP7* and *GIMAP8* in 62 RNAseq libraries from 18 healthy women without BC *versus* 16 women with ductal carcinoma in situ (DCIS), 15 with HER2-positive (HER2+) and 13 triple-negative (TNeg). Note the significantly downregulated mRNA expression in the BC tissue, compared with the levels of expression in DCIS breast tissue. **Log2FC**: Logarithm of fold change.

The RNAseq libraries used were from the study of Varley et al. [14]:

Healthy women: SRR2040339, SRR2040340, SRR2040341, SRR2040342, SRR2040343, SRR2040344, SRR2040345, SRR2040346, SRR2040347, SRR2040348, SRR3151763, SRR1313204, SRR1313205, SRR1313206, SRR1313207, SRR1313208, ERR358485, ERR358486

Women with DCIS: SRR2040369, SRR2040370, SRR2040371, SRR2040372, SRR2040373, SRR1822176, SRR1822182, SRR1822177, SRR1822183, SRR1822178, SRR1822184, SRR1822179, SRR1822185, SRR1822180, SRR1822186, SRR1822187

Women with HER2+ BC: SRR1538692, SRR1538693, SRR1538694, SRR1538695, SRR1538696, SRR1538697, SRR1538698, SRR1538699, SRR1538700, SRR1600265, SRR1600266, SRR1600267, SRR1600268, SRR1600270, SRR1600271

Women TNeg BC: SRR1313132, SRR1313133, SRR1313134, SRR1313135, SRR1313136, SRR1313137, SRR1313148, SRR1313147, SRR1313146, SRR1313145, SRR1313144, SRR1313143, SRR1313142
